# Prevalence and distribution patterns of drug resistance in *Mycobacterium tuberculosis* to first-line antituberculosis drugs in Urumqi, China

**DOI:** 10.1128/spectrum.01370-25

**Published:** 2025-09-03

**Authors:** Jiandong Yang, Pengwei Lou, Weisheng Zhang, Yanggui Chen, Yaoqin Lu, Na Xue, Peisheng Wang, Kai Wang

**Affiliations:** 1 Department of Tuberculosis Control and Prevention, Urumqi Center for Disease Control and Prevention, Urumqi, People’s Republic of China; 2 School of Public Health, Xinjiang Medical University, Urumqi, People’s Republic of China; 3 Department of Big Data, College of Information Engineering, Xinjiang Institute of Engineering, Urumqi, People’s Republic of China; 4 Department of Infectious Disease Control and Prevention, Urumqi Center for Disease Control and Prevention, Urumqi, People’s Republic of China; 5 Department of Science, Education and International Exchange, Urumqi Center for Disease Control and Prevention, Urumqi, People’s Republic of China; 6 Laboratory of Pathogenesis, Prevention and Treatment of High Incidence Diseases in Central Asia, Department of Medical Engineering and Technology, Xinjiang Medical University, Urumqi, China; Petrified Bugs LLC, Miami, Florida, USA

**Keywords:** tuberculosis, drug-resistant tuberculosis, distribution characteristics

## Abstract

This study aimed to investigate the epidemic status and distribution characteristics of drug-resistant *Mycobacterium tuberculosis* in Urumqi. From January 2019 to July 2024, all sputum culture-positive *Mycobacterium tuberculosis* strains were collected in Urumqi. Using the traditional solid-state proportion method for drug-sensitivity testing, we determined the resistance of *Mycobacterium tuberculosis* to first-line antituberculosis (TB) drugs. The epidemic status and distribution characteristics of first-line antituberculosis drug-resistant *Mycobacterium tuberculosis* were analyzed using R statistical software (version 3.6.1). Among the 1,241 culture-positive *Mycobacterium tuberculosis* strains included in this analysis, 973 (78.40%) were smear positive. The overall proportion of non-tuberculous mycobacteria was 2.5%. The overall prevalence of drug-resistant tuberculosis (DR-TB) was 18.93%, with a prevalence of 17.78% in new cases and 24.40% in retreatment cases, respectively. In this survey, the overall prevalence was 10.91% (132 out of 1,210) for mono-drug-resistant tuberculosis, 3.72% (45 out of 1,210) for polydrug-resistant tuberculosis, and 4.30% (52 out of 1,210) for multidrug-resistant tuberculosis. Moreover, the patterns of resistance to first-line anti-TB drugs were highly diverse. The drug-resistance rate among retreatment tuberculosis patients in Urumqi remains notably high. Thus, enhancing drug-resistance surveillance in these patients is critical for effective tuberculosis prevention and control in the region.

**IMPORTANCE:**

The result of this study indicated that DR-TB is a serious public health problem in Urumqi. Resistance drugs distributed type to first-line anti-TB drugs are very broad in Urumqi. Any resistance to anti-TB drugs in new cases is more than in retreatment cases.

## INTRODUCTION

Tuberculosis (TB) has emerged as a critical public health challenge affecting millions globally (1). As a persistent infectious disease, it continues to pose a significant threat to human health (2). The *Global Tuberculosis Report 2024* estimates that in 2023, there were 10.8 million new TB cases and 1.25 million TB-related deaths worldwide, with an incidence rate of 134 cases per 100,000 population (3). TB management is complicated by suboptimal treatment outcomes, particularly for drug-resistant tuberculosis (DR-TB) (4, 5).

The emergence of DR-TB at the end of the 20th century not only exacerbated the global TB epidemic but also introduced unprecedented challenges to tuberculosis control and prevention efforts (6, 7). In 2023, an estimated 400,000 incident cases of multidrug-resistant/rifampicin-resistant tuberculosis (MDR/RR-TB) occurred worldwide, with China accounting for 29,000 cases (corresponding to a rate of 2.1 cases per 100,000 population) (3). Studies indicate that DR-TB incidence is influenced by multiple factors, including mycobacterial drug-resistance gene mutations, prior treatment history, and treatment adherence (8, 9). Additionally, increased global population mobility has facilitated the sustained transmission of DR-TB. Clinically, DR-TB management is characterized by prolonged treatment durations, complex regimens, suboptimal outcomes, and high costs (10, 11). Thus, understanding anti-TB drug-resistance patterns—especially among newly diagnosed cases—is essential for designing effective TB control strategies (12).

China is among the countries with the highest burdens of TB and MDR/RR-TB, ranking fourth globally in MDR/RR-TB cases (comprising 7.3% of the worldwide total) (3). TB prevalence in Western China exceeds that in central and eastern regions, with Xinjiang Uygur Autonomous Region reporting some of the highest TB and MDR/RR-TB incidence rates nationally (13). According to *The Fifth Tuberculosis Sampling Survey Report of Xinjiang*, the drug-resistance rate of TB in Xinjiang is as high as 34.88%, among which the drug-resistance rates of newly treated patients and retreated patients are 31.94% and 50.00%, respectively (14). The overall TB drug-resistance rate in Xinjiang was essentially comparable to the national average (36.80%) (14, 15). The drug-resistance rate in newly treated patients was lower than the national level (36.90%), but the drug-resistance rate in retreated patients was higher than the national level (35.90%) (14, 15). As the capital of Xinjiang, Urumqi has observed an upward trend in *Mycobacterium tuberculosis* (MTB) drug resistance, according to surveillance by the Urumqi Center for Disease Control and Prevention’s National Tuberculosis Reference Laboratory. This cross-sectional study aims to characterize the distribution of MTB resistance to first-line anti-TB drugs, providing evidence-based insights to inform local DR-TB prevention and control strategies.

## MATERIALS AND METHODS

### Study design

This study is a cross-sectional research based on drug-resistance surveillance of MTB, conducted at the Tuberculosis Reference Laboratory of Urumqi Center for Disease Control and Prevention (UCDC). We retrospectively analyzed the distribution of MTB resistance to first-line antitubercular drugs in Urumqi from 2019 to 2024. UCDC serves as the agency responsible for monitoring MTB drug resistance across the entire city. All sputum culture-positive MTB strains were sent to the Tuberculosis Reference Laboratory of UCDC by hospitals in Urumqi. Basic information of TB patients, such as age, sex, and occupation, was obtained from the China Information System of Disease Prevention and Control.

### Sample size

The study population consisted of all TB patients with sputum culture-positive results in Urumqi from January 2019 to July 2024, and the total number of these patients was 1,241.

### Experimental methods

The experimental methods used in this study included sputum smear examination, mycobacterial isolation and culture, species identification, and drug susceptibility testing (DST). All the abovementioned experimental methods were strictly performed in accordance with the procedural standards outlined in the *Technical Guidelines for Tuberculosis Prevention and Control in China* (https://www.chinacdc.cn/jkyj/crb2/yl/fjh/jswj_fjh/202410/P020241010432930570191.pdf). Detailed experimental and quality control procedures are described in Appendices S1–S4. The reagents and consumables utilized were supplied by Zhuhai Baso Biotechnology Co., Ltd. Throughout the testing period, the laboratory successfully passed the external quality assessment conducted by the National Center for Tuberculosis Control and Prevention, China Centers for Disease Control and Prevention.

### Biosafety

According to the *Catalogue of Pathogenic Microorganisms Transmissible to Humans* (2023 Edition) (https://www.gkgzj.com/u/cms/www/202309/13101321cj0r.pdf) promulgated by China’s National Health Commission, MTB is categorized as a Category II pathogenic microorganism. Given the potential biohazard risks inherent in the experimental procedures, we strictly adhere to the biosafety requirements for MTB experimental manipulations outlined in both the *Catalogue of Pathogenic Microorganisms Transmissible to Humans* (2023 Edition) and the *Technical Guidelines for Tuberculosis Prevention and Control in China*. Specifically, smear tests, culture experiments, strain identification tests, and drug-sensitivity tests for MTB were conducted in enhanced Biosafety Level 2 laboratories, utilizing biosafety facilities and equipment such as biological safety cabinets, autoclaves, and personal protective equipment, including N95 masks, positive-pressure protective headgear, biosafety protective suits, and other relevant gear. All experimental personnel have undergone specialized biosafety training and operational training and obtained the necessary qualifications to perform these procedures.

### Data analysis

The data were entered into Microsoft Excel 2010 and subsequently analyzed using R (version 3.6.1). Frequencies and percentages were employed to describe the data.

## RESULTS

### Sociodemographic characteristics

A total of 1,241 culture-positive MTB isolates were sent to the National Tuberculosis Reference Laboratory of the UCDC for DST between January 2019 and July 2024. During this period, 1,029 (82.92%) of the tested MTB isolates were from new TB cases, while 212 (17.08%) were from retreatment cases. Age distribution analysis revealed that the largest proportion of TB patients (28.53%) undergoing DST fell within the 20–35 years age group, followed by those aged ≥65 years (25.46%). Males constituted 66.32% of the study population, exceeding females at 33.68%. In terms of demographic characteristics, non-retirees, non-floating populations, and individuals without diabetes outnumbered retirees, floating populations, and those with diabetes, respectively. Of the 1,241 culture-positive MTB cases, 128 did not provide HIV test results. Among the remaining 1,113 cases with available data, 1,069 (86.14%) tested negative for HIV, and 44 (3.55%) tested positive (Table 1).

**TABLE 1:** Sociodemographic characteristics of the included study population

Characteristics	New cases (*n* = 1,029 [82.92%])		Retreatment cases (*n* = 212 [17.08%])		Total (*n* = 1,241 [100.00%])	
	*n*	%	*n*	%	*n*	%
Age						
<20	43	4.18	4	1.89	47	3.79
20–35	306	29.74	48	22.64	354	28.53
35–50	233	22.64	62	29.25	295	23.77
50–65	190	18.46	39	18.40	229	18.45
≥65	257	24.98	59	27.83	316	25.46
Sex						
Male	676	65.69	147	69.34	823	66.32
Female	353	34.31	65	30.66	418	33.68
Farmer						
No	923	89.70	197	92.92	1,120	90.25
Yes	106	10.30	15	7.08	121	9.75
Retiree						
No	849	82.51	182	85.85	1,031	83.08
Yes	180	17.49	30	14.15	210	16.92
Floating population						
No	648	62.97	141	66.51	789	63.58
Yes	381	37.03	71	33.49	452	36.42
Diabetes						
No	926	89.99	183	86.32	1,109	89.36
Yes	103	10.01	29	13.68	132	10.64
HIV						
Not provided	118	11.47	10	4.72	128	10.31
Negative	881	85.62	188	88.68	1,069	86.14
Positive	30	2.92	14	6.60	44	3.55

### Smear and bacterial type

Of the total smears performed, 973 (78.40%) were positive and 268 (21.60%) were negative. Smear-positive results were more prevalent than negative results in both new cases and retreatment cases and the overall cohort. Non-tuberculous mycobacteria (NTMs) comprised 2.50% (31 out of 1,241) of total cases, 2.72% (28 out of 1,029) of new cases, and 1.42% (3 out of 212) of retreatment cases, respectively (Table 2).

**TABLE 2:** Characteristics of smear and bacterial types in the included study population

Status	New cases (*n* = 1,029 [82.92%])		Retreatment cases (*n* = 212 [17.08%])		Total (*n* = 1,241 [100.00%])	
	*n*	% (95% CI)	*n*	% (95% CI)	*n*	% (95% CI)
Smear result						
Negative	255	24.78 (22.18–27.49)	13	6.13 (3.05–9.52)	268	21.60 (19.34–24.01)
Positive	774	75.22 (72.51–77.82)	199	93.87 (90.48–96.95)	973	78.40 (75.99–80.66)
Bacterial type						
TB	1,001	97.28 (96.31–98.22)	209	98.58 (96.65–100.00)	1,210	97.50 (96.62–98.31)
NTB	28	2.72 (1.78–3.69)	3	1.42 (0.00–3.35)	31	2.50 (1.69–3.38)

### First-line antituberculosis drug resistance state

**Fig 1:**
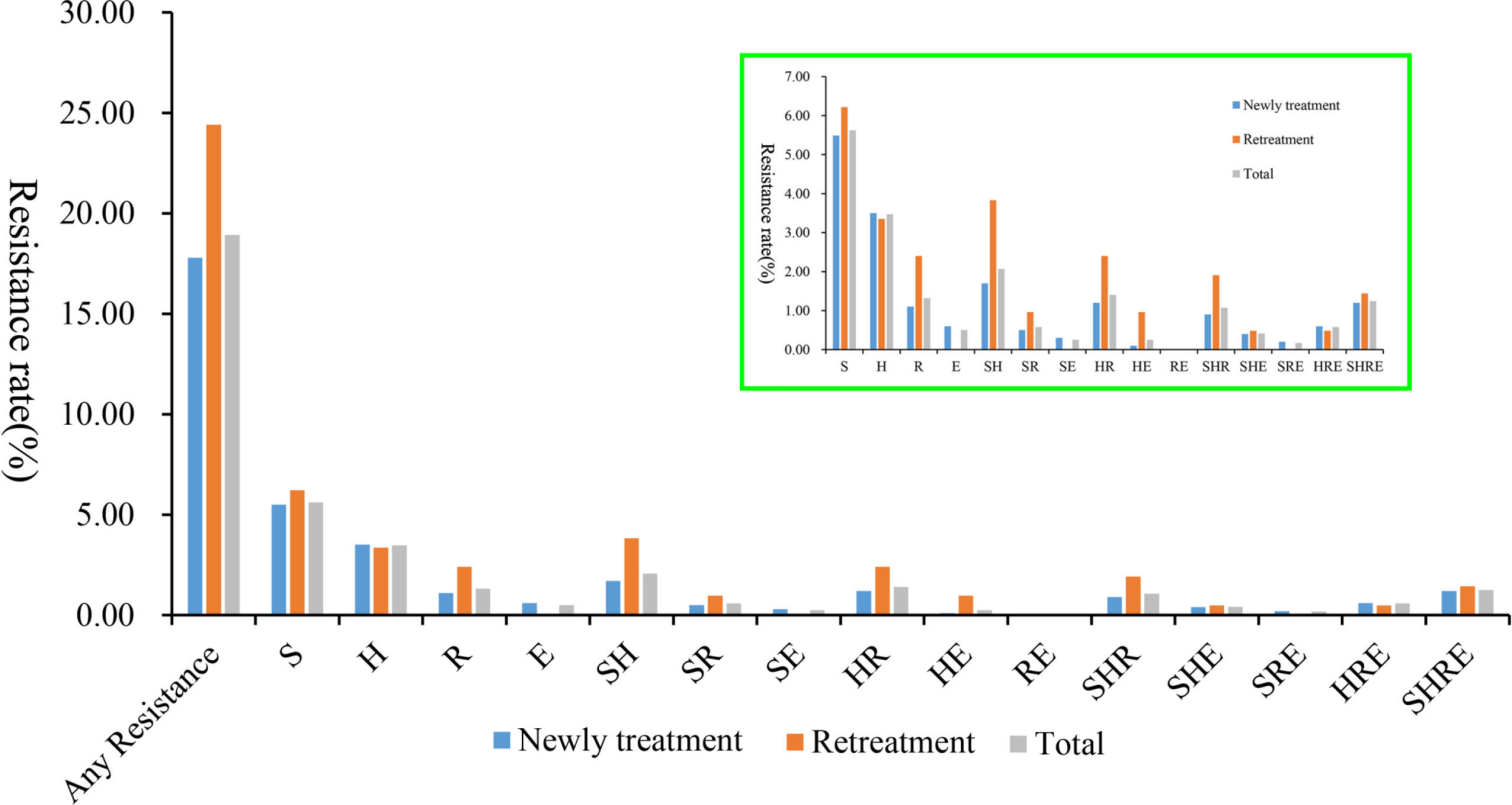
Distribution map of drug-resistance profiles to first-line antitubercular drugs. The *X*-axis represents resistance combinations, and the *Y*-axis represents resistance rates. The distribution of resistance rates for the remaining resistance profiles is shown within the green box in the figure, excluding the resistance rate to any single antitubercular drug.

Table 3 and Fig. 1 illustrate the distribution of resistance to first-line antituberculosis drugs among 1,210 MTB isolates. Of these, 229 (18.93%) exhibited resistance to at least one first-line drug (isoniazid [H], rifampicin [R], ethambutol [E], or streptomycin [S]). The overall prevalence was 10.91% (132 out of 1,210) for mono-drug-resistant TB, 3.72% (45 out of 1,210) for polydrug-resistant TB, and 4.30% (52 out of 1,210) for multidrug-resistant tuberculosis (MDR-TB). Among cases with resistance to at least one first-line drug, mono-drug-resistant, polydrug-resistant, and MDR-TB accounted for 57.64% (132), 19.65% (45), and 22.71% (52), respectively. The most common patterns were mono-drug resistance to streptomycin (5.62%), polydrug resistance to streptomycin and isoniazid (S + H, 2.07%), and MDR-TB (H + R, 1.40%). In stratified analysis by patient type, 178 of 1,001 new cases (17.78%) and 51 of 209 retreatment cases (24.40%) had resistance to at least one first-line drug. In new cases, mono-drug-resistant, polydrug-resistant, and MDR-TB rates were 10.69% (107 out of 1,001), 3.20% (32 out of 1,001), and 3.90% (39 out of 1,001), respectively, comprising 60.11%, 17.98%, and 21.91% of resistant cases. The predominant resistance patterns were mono-drug resistance to streptomycin (5.49%), polydrug resistance to streptomycin and isoniazid (1.70%), and MDR-TB (1.20% including H + R and H + R + E/S). Retreatment cases showed a similar resistance pattern but higher overall resistance (24.40% vs 17.78% in new cases). Their mono-drug-resistant, polydrug-resistant, and MDR-TB rates were 11.96% (25 out of 209), 6.22% (13 out of 209), and 6.22% (13 out of 209), respectively, with equal proportions of polydrug-resistant and MDR-TB rates.

**TABLE 3:** Drug susceptibility pattern of MTB to first-line anti-TB drugs^*a*^

Resistance status	New cases (*n* = 1,001 [82.73%])		Retreatment cases (*n* = 209 [17.27%])		Total (*n* = 1,210 [100.00%])	
	*n*	% (95% CI)	*n*	% )95% CI)	*n*	% (95% CI)
Total susceptible	823	82.22 (79.98–84.58)	158	75.60 (69.80–81.10)	981	81.07 (78.84–83.22)
Any resistance	178	17.78 (15.42–20.02)	51	24.40 (18.90–30.20)	229	18.93 (16.78–21.16)
Resistance to S	55	5.49 (4.16–6.89)	13	6.22 (3.21–9.80)	68	5.62 (4.38–6.86)
Resistance to H	35	3.50 (2.39–4.57)	7	3.35 (1.09–5.93)	42	3.47 (2.48–4.63)
Resistance to R	11	1.10 (0.50–1.81)	5	2.40 (0.49–4.71)	16	1.32 (0.66–2.07)
Resistance to E	6	0.60 (0.20–1.08)	0	0.00 (0.00–0.00)	6	0.50 (0.17–0.91)
Resistance to SH	17	1.70 (1.00–2.56)	8	3.83 (1.42–6.60)	25	2.07 (1.24–2.89)
Resistance to SR	5	0.50 (0.10–0.99)	2	0.96 (0.00–2.51)	7	0.58 (0.17–0.99)
Resistance to SE	3	0.30 (0.00–0.70)	0	0.00 (0.00–0.00)	3	0.25 (0.00–0.58)
Resistance to HR	12	1.20 (0.59–1.92)	5	2.40 (0.49–4.71)	17	1.40 (0.74–2.15)
Resistance to HE	1	0.10 (0.00–0.30)	2	0.96 (0.00–2.51)	3	0.25 (0.00–0.58)
Resistance to RE	0	0.00 (0.00–0.00)	0	0.00 (0.00–0.00)	0	0.00 (0.00–0.00)
Resistance to SHR	9	0.90 (0.40–1.58)	4	1.91 (0.46–4.17)	13	1.07 (0.50–1.65)
Resistance to SHE	4	0.40 (0.10–0.89)	1	0.48 (0.00–1.55)	5	0.41 (0.08–0.83)
Resistance to SRE	2	0.20 (0.00–0.50)	0	0.00 (0.00–0.00)	2	0.17 (0.00–0.41)
Resistance to HRE	6	0.60 (0.20–1.08)	1	0.48 (0.00–1.55)	7	0.58 (0.17–1.07)
Resistance to SHRE	12	1.20 (0.59–1.92)	3	1.44 (0.00–3.35)	15	1.24 (0.66–1.90)

^*a*^ CI, confidence interval; E, ethambutol; H, isoniazid; HE, isoniazid and ethambutol; HR, isoniazid and rifampicin; HRE, isoniazid, rifampicin, and ethambutol; *n*, number of samples; R, rifampicin; RE, rifampicin and ethambutol; S, streptomycin; SE, streptomycin and ethambutol; SH, streptomycin and isoniazid; SHE, streptomycin, isoniazid, and ethambutol; SHR, streptomycin, isoniazid, and rifampicin; SHRE, streptomycin, isoniazid, rifampicin, and ethambutol; SR, streptomycin and rifampicin; SRE, streptomycin, rifampicin, and ethambutol.

### Analysis of the trends in resistance to first-line antituberculosis drugs and associated influencing factors

**Fig 2:**
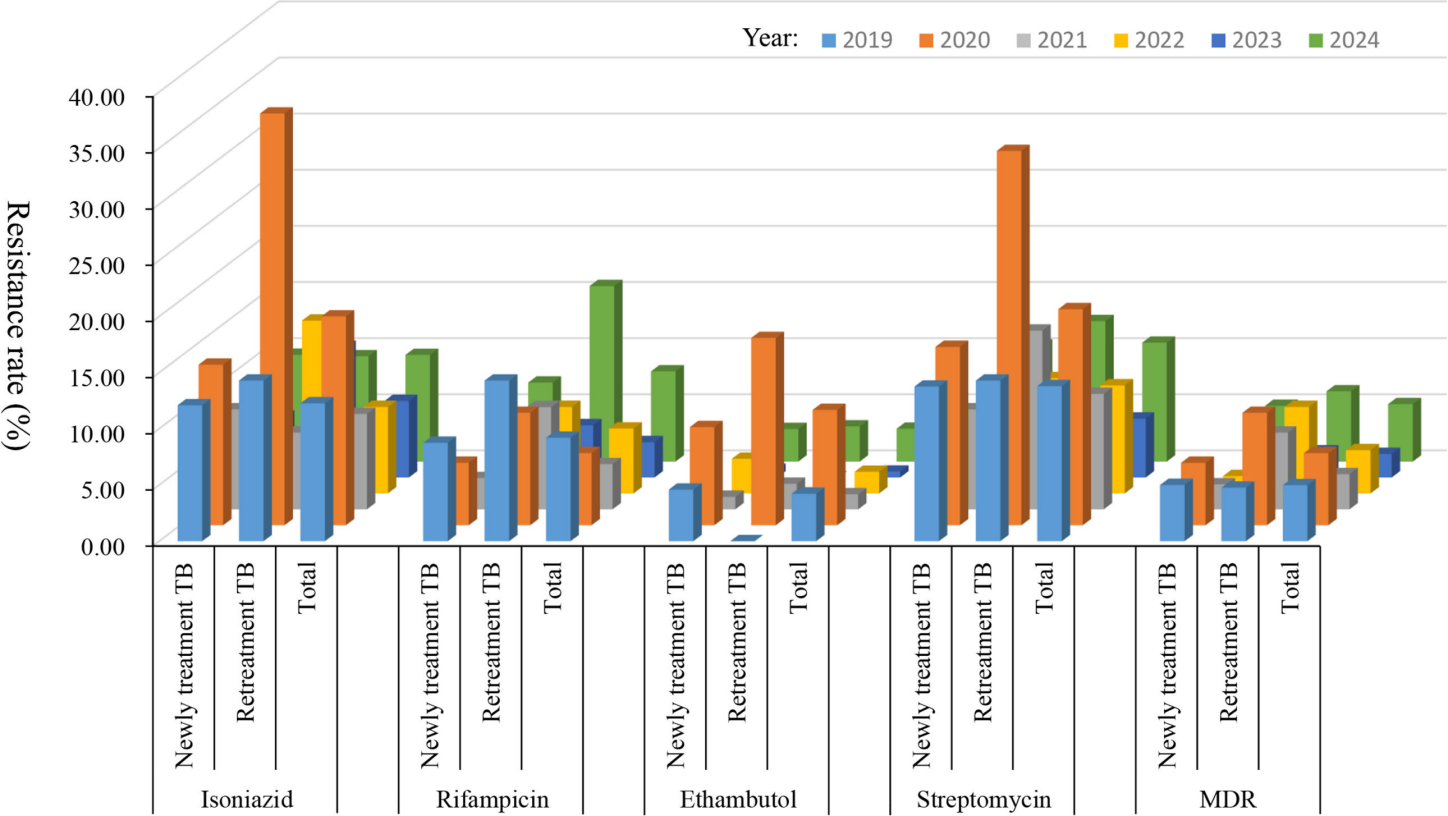
The distribution of drug-resistance rates of *Mycobacterium tuberculosis* isolated from tuberculosis patients in different treatment categories to isoniazid, rifampicin, ethambutol, streptomycin, and multidrug-resistant (MDR) from 2019 to 2024. The *X*-axis represents different treatment categories for isoniazid, rifampicin, ethambutol, streptomycin, and multidrug resistance. The *Y*-axis denotes drug-resistance rates, and the *Z*-axis indicates time (2019–2024).

**Fig 3:**
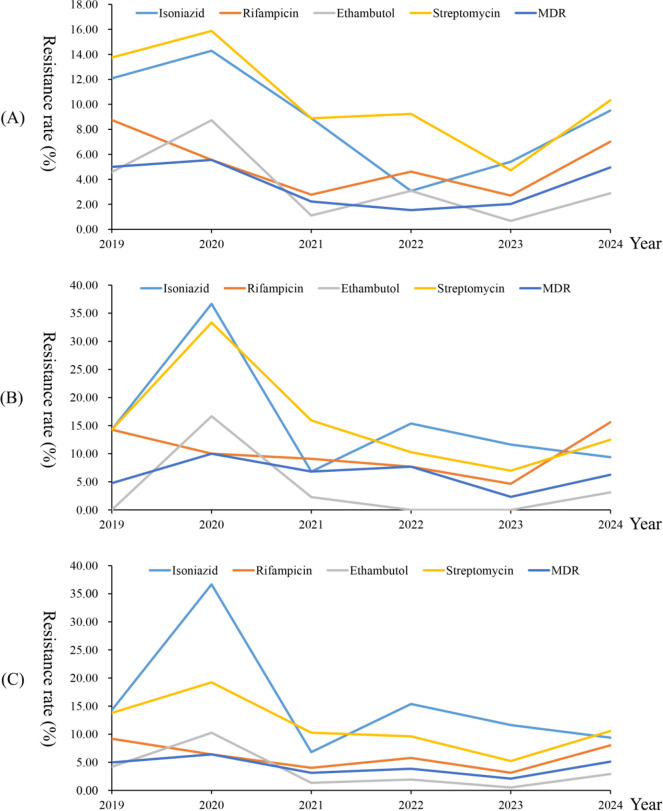
The temporal distribution of drug-resistance rates of *Mycobacterium tuberculosis* to isoniazid, rifampicin, ethambutol, streptomycin, and multidrug-resistant (MDR). (**A**) The temporal distribution of drug-resistance rates among *Mycobacterium tuberculosis* isolates from newly treated patients to isoniazid, rifampicin, ethambutol, streptomycin, and MDR. (**B**) The temporal distribution of drug-resistance rates among *Mycobacterium tuberculosis* isolates from retreatment patients to isoniazid, rifampicin, ethambutol, streptomycin, and MDR. (**C**) The temporal distribution of drug-resistance rates among *Mycobacterium tuberculosis* isolates from total patients to isoniazid, rifampicin, ethambutol, streptomycin, and MDR.

Using a χ^2^ test for trend, we observed significant trends in the resistance rates to isoniazid, ethambutol, and streptomycin (all *P* values of <0.05). Newly treated patients also exhibited significant trends in resistance rates to isoniazid, ethambutol, and streptomycin (all *P* values of <0.05), whereas only streptomycin resistance showed a significant trend in retreated patients (*P* = 0.0447). Inspection of trend plots revealed that all observed trends were downward. Notably, no significant trends were observed for multidrug-resistance rates in MTB (neither for overall multidrug-resistance rates nor for those stratified by newly treated and retreated patients). Detailed results are presented in Table 4 and Fig. 2 and 3.

**TABLE 4:** Drug susceptibility pattern of MTB to first-line anti-TB drugs^*a*^

Group	2019 (*N* = 261)		2020 (*N* = 156)		2021 (*N* = 224)		2022 (*N* = 104)		2023 (*N* = 191)		2024 (*N* = 274)		χ^2^	*P*
	S	R	S	R	S	R	S	R	S	R	S	R		
Isoniazid														
Newly treated TB	211	29	108	18	164	16	63	2	140	8	219	23	3.8535	0.0496
Retreatment TB	18	3	19	11	41	3	33	6	38	5	29	3	3.0909	0.0787
Total	229	32	127	29	205	19	96	8	178	13	248	26	5.6156	0.0178
Rifampicin														
Newly treated TB	219	21	119	7	175	5	62	3	144	4	225	17	1.0439	0.3069
Retreatment TB	18	3	27	3	40	4	36	3	41	2	27	5	0.0359	0.8496
Total	237	24	146	10	215	9	98	6	185	6	252	22	0.7916	0.3736
Ethambutol														
Newly treated TB	229	11	115	11	178	2	63	2	147	1	235	7	4.8874	0.0271
Retreatment TB	21	0	25	5	43	1	39	0	43	0	31	1	2.9645	0.0851
Total	250	11	140	16	221	3	102	2	190	1	266	8	7.1308	0.0076
Streptomycin														
Newly treated TB	207	33	106	20	164	16	59	6	141	7	217	25	5.1021	0.0239
Retreatment TB	18	3	20	10	37	7	35	4	40	3	28	4	4.0297	0.0447
Total	225	36	126	30	201	23	94	10	181	10	245	29	7.5989	0.0058
MDR														
Newly treated TB	228	12	119	7	176	4	64	1	145	3	230	12	0.3285	0.5665
Retreatment TB	20	1	27	3	41	3	36	3	42	1	30	2	0.3595	0.5488
Total	248	13	146	10	217	7	100	4	187	4	260	14	0.4756	0.4904

^*a*^ S, sensitivity; R, resistance. χ^2^ values represent χ^2^ for trend, and the reported *P* values are from the trend test.

Influencing factor analysis of resistance rates to first-line antituberculosis drugs and multidrug resistance was performed with age, gender, occupation (farmer or not), retirement status, migrant population status, diabetes mellitus history, HIV test results, treatment category, and sputum smear results as covariates. Multivariable logistic regression analysis showed good model fit (all Hosmer-Lemeshow goodness-of-fit test *P* values of >0.05). Treatment category was identified as a significant predictor for resistance to isoniazid, rifampicin, and streptomycin. Retreatment status emerged as a risk factor for resistance to these drugs (all *P* values of <0.05), with odds ratios and 95% confidence intervals (CIs) of 1.6977 (1.0774–2.6750), 1.8886 (1.0789–3.3060), and 1.5779 (1.0059–2.4752), respectively. Notably, none of the covariates examined were significant predictors for multidrug resistance or ethambutol resistance. Detailed results are provided in Appendix S5.

## DISCUSSION

MDR/RR-TB represents one of the most severe public health challenges globally. China is among the nations significantly affected by MDR/RR-TB. Between 2010 and 2011, the Chinese National Center for Tuberculosis Control and Prevention conducted an investigation on the prevalence of tuberculosis in China. The findings of this investigation revealed that the resistance rates of MTB to first-line antituberculosis drugs were 36.80% (95% CI: 31.10%–42.70%), and the multidrug-resistance rate of MTB was 6.80% (95% CI: 4.10%–10.40%) (15). Urumqi, the capital city of Xinjiang Uygur Autonomous Region in Western China, faces an unoptimistic situation regarding the epidemic of DR-TB. Despite the escalating burden of MTB resistance to first-line antituberculosis drugs in Urumqi, there is a paucity of information derived from routine laboratory data. Therefore, this study aimed to characterize the distribution of MTB resistance to first-line antituberculosis drugs among patients in Urumqi.

Culture data from 1,241 culture-positive MTB strains were reviewed. During the study period, smear microscopy revealed a positivity rate of 78.40% (973 out of 1,241) among these strains. NTMs were identified in 31 cases (2.50%). DST for first-line anti-tuberculosis drugs was conducted on 1,210 MTB isolates. Of these, 981 (81.07%) were susceptible to all tested drugs, while 229 (18.93%) exhibited resistance to at least one first-line agent. The overall prevalence was 10.91% (132 out of 1,210) for mono-drug-resistant TB, 3.72% (45 out of 1,210) for polydrug-resistant TB, and 4.30% (52 out of 1,210) for MDR-TB. In stratified analysis, new cases (*n* = 1,001) showed prevalences of 10.69% (107 out of 1,001) for mono-drug-resistant TB, 3.20% (32 out of 1,001) for polydrug-resistant TB, and 3.90% (39 out of 1,001) for MDR-TB. Retreatment cases (*n* = 209) had higher rates: 11.96% (25 out of 209) for mono-drug-resistant TB, 6.22% (13 out of 209) for polydrug-resistant TB, and 6.22% (13 out of 209) for MDR-TB.

The overall prevalence of DR-TB in this survey was lower than that reported in previous national survey data, while it was approximately similar to the data reported by Peng et al. (15, 16). Numerous studies in China have shown that the rates of TB drug resistance vary across different regions of the country (15–20). This variation is primarily associated with differences in TB-related preferential policies and economic levels among different regions. Additionally, the varying frequencies of population mobility in different parts of China also contribute to the disparities in TB resistance rates. The frequency of population mobility is positively correlated with the risk of DR-TB spread. Given that the frequency of population mobility in Eastern China is higher than that in Western China, the TB resistance rates in Eastern China are also higher. Compared with the central and eastern regions, Xinjiang (including Urumqi) in the western region exhibits lower population mobility and a relatively narrower scope of population movement (21). This phenomenon is unfavorable for the transmission of drug-resistant MTB, thereby leading to relatively lower overall drug resistance of MTB. However, studies also show that population mobility in Xinjiang, including Urumqi, is accelerating, which suggests that the drug-resistance rate of MTB in Urumqi may face an upward risk in the future. Additionally, differences in treatment adherence among tuberculosis patients may also contribute to variations in drug-resistance rates of MTB, with regions showing low treatment adherence often having higher drug-resistance rates (22, 23). According to our previous monitoring results and research findings by relevant scholars, TB patients in Xinjiang (including Urumqi) have relatively high treatment adherence (24). Such relatively high treatment adherence significantly reduces the drug resistance of MTB. It is worth noting that the Xinjiang model for TB prevention and control has also played a significant role in reducing the drug-resistance rate of MTB (25). The Xinjiang model for TB prevention and control can be summarized as maximizing patient detection, expanding the coverage of intervention efforts to the greatest extent, and maximizing the improvement of treatment quality (26). This model is an effective approach to controlling tuberculosis epidemics and a concrete manifestation of implementing the TB prevention and control mechanism of “government leadership, each department assuming its respective responsibilities, and wide participation of the whole society” (26). Under this model, drug-resistant patients can be rapidly identified and subjected to centralized isolation treatment, which greatly reduces the risk of transmission of drug-resistant MTB. The lower drug-resistance rate of MTB in Urumqi compared to the national level may benefit from this. Furthermore, our previous research results indicate that the lineage distribution of MTB in Urumqi differs from that in other provinces (27). Studies have shown that drug resistance varies among different lineages of MTB (28). In summary, the reasons for the differences in drug-resistance rates of MTB between Urumqi and the national level are diverse, and the specific causes still require further exploration.

In this study, mono-resistance to S was the most prevalent pattern of mono-drug-resistant TB across all patient groups (new cases, retreatment cases, and the overall cohort). We hypothesize that this observation may be linked to the genetic background of MTB circulating in Urumqi. Notably, the Beijing lineage constitutes the predominant genotype in this region, and numerous studies have shown that Beijing lineage isolates are frequently associated with mutations in the *rpsL* gene at codon 43, which confers streptomycin resistance (29–31). Polydrug resistance most commonly involved H and S across all patient subgroups. However, the predominant patterns of MDR-TB differed by patient type: in new cases, MDR-TB primarily involved resistance to isoniazid and rifampicin (H + R), often with additional resistance to streptomycin and ethambutol (H + R + S + E), whereas retreatment cases and the overall cohort showed a higher proportion of classic H + R MDR-TB without additional drug resistance. These findings highlight the considerable diversity in first-line anti-TB drug-resistance patterns in Urumqi, a characteristic that complicates efforts to implement standardized prevention and control strategies for drug-resistant TB.

Additionally, we observed a downward trend in the resistance rates of certain first-line antitubercular drugs. We hypothesize that this phenomenon may be associated with heightened protective awareness among the population following the coronavirus disease 2019 (COVID-19) pandemic. Increased adoption of preventive measures (particularly mask-wearing) likely inhibits the transmission of drug-resistant MTB. Studies have shown a decline in the incidence of respiratory infectious diseases (including TB) after the COVID-19 pandemic (32, 33), which may provide a plausible explanation for the observed decrease in MTB resistance rates. Although resistance rates have decreased, existing research indicates that this decline is likely transient (34); thus, continued vigilance regarding MTB drug resistance remains imperative. We also observed that retreatment was a risk factor for resistance to isoniazid, rifampicin, and streptomycin. This finding aligns with previous research by numerous scholars (35, 36). This highlights the need for increased vigilance regarding drug resistance when clinically managing retreatment tuberculosis patients, emphasizing the importance of selecting drugs based on drug-sensitivity test results. In this study, no influencing factors for multidrug resistance in *Mycobacterium tuberculosis* were identified. To address this, future tuberculosis drug-resistance surveillance will prioritize investigating related factors to actively explore potential associations.

Since this study is a cross-sectional analysis utilizing drug-resistance surveillance data of MTB, certain limitations exist. Although we conducted a time-trend analysis of annual drug-resistance rates, the evidence level remains relatively low and requires validation by follow-up studies. Additionally, as the drug susceptibility testing in this study employed phenotypic methods, only culture-positive MTB isolates were included, which may introduce some bias. To more comprehensively and authentically reflect the drug-resistance profile of MTB in Urumqi, future studies could utilize molecular biological methods (such as whole-genome sequencing, gene chips, and probe technology) to analyze drug resistance in culture-negative but smear-positive MTB strains. Additionally, according to the *Technical Guidelines for Tuberculosis Prevention and Control in China*, TB patients in Urumqi typically receive antitubercular treatment with fixed-dose combination preparations, which are primarily composed of first-line antitubercular drugs. Second-line and third-line antitubercular drugs are only used for treatment during individualized dosing in special circumstances such as detected drug resistance or adverse reactions, but such cases are relatively rare. Therefore, this study only analyzed the drug-resistance status of first-line antitubercular drugs and did not consider the drug-resistance status of second-line and third-line antitubercular drugs. This limitation resulted in an inability to assess the distribution of pre-extensively drug-resistant and extensively drug-resistant tuberculosis, which have garnered significant attention recently. In light of this, in subsequent research, we will further investigate the drug resistance of second-line and third-line antitubercular drugs and maintain continuous and comprehensive attention to the drug-resistance status of MTB.

### Conclusion

This study highlights substantial diversity in DR-TB resistance patterns in Urumqi. The overall prevalence of DR-TB was 18.93%, with resistance rates of 17.78% and 24.40% observed in new and retreatment cases, respectively. Strengthening drug-resistance prevention initiatives, control programs, and surveillance systems is critical to mitigating the disease burden in the region. In future work, drug-resistance surveillance of MTB should be strengthened, particularly among retreatment TB patients. Additionally, health education regarding medication adherence should be enhanced for retreatment TB patients. Furthermore, efforts should be made to further refine and sustain the implementation of the Xinjiang model for TB prevention and control.

## Data Availability

Data that support the findings of this study are available from corresponding author on reasonable request and with permission from the corresponding author.

## SUPPLEMENTAL MATERIAL

Supplemental materials spectrum.01370-25-s0001.docx Experimental methods and supplemental data analysis tables.

ASM does not own the copyrights to Supplemental Material that may be linked to, or accessed through, an article. The authors have granted ASM a non-exclusive, world-wide license to publish the Supplemental Material files. Please contact the corresponding author directly for reuse.
