# Supplementary material for: Prevalence and distribution patterns of drug resistance in Mycobacterium tuberculosis to first-line antituberculosis drugs in Urumqi, China
Source: Microbiol Spectr. 2025 Sep 3;13(10):e01370-25. doi: 10.1128/spectrum.01370-25 (PMC12502695; doi:10.1128/spectrum.01370-25)
Supplement: Supplemental materials — Experimental methods and supplemental data analysis tables. [file spectrum.01370-25-s0001.docx]

**Appendix**

**Table of Contents**

[**Appendix S1 Sputum Smear Experimental Method** 2](#_Toc199804732)

[S1.1 *Preparation of Sputum Smears* 2](#_Toc199804733)

[S1.2 *Staining* 2](#_Toc199804734)

[S1.3 *Microscopic Examination* 3](#_Toc199804735)

[S1.4 *Criteria for Result Interpretation* 4](#_Toc199804736)

[**Appendix S2 Experimental methods for the isolation and cultivation of bacterial strains** 5](#_Toc199804737)

[S2.1 *Mycobacterium isolation and culture steps* 5](#_Toc199804738)

[S2.2 *Results reading* 6](#_Toc199804739)

[**Appendix S3 Experimental methods for the identification of microbial strains** 8](#_Toc199804740)

[S3.1 *Test procedures* 8](#_Toc199804741)

[S3.2 *Identification criteria* 9](#_Toc199804742)

[**Appendix S4 Antimicrobial susceptibility testing methods** 11](#_Toc199804743)

[S4.1 *Test procedures* 11](#_Toc199804744)

[S4.2 *Results reading and resistance determination criteria* 14](#_Toc199804745)

[S4.3 *Quality control* 14](#_Toc199804746)

[**Appendix S5 Supplementary results** 16](#_Toc199804747)

[S5.1 *Logistic Regression Model Variable Assignment Table* 16](#_Toc199804748)

[S5.2 The Hosmer- Lemeshow goodness-of-fit test result of the logistic regression 16](#_Toc199804749)

[S5.3 The results of the multivariate logistic regression analysis 17](#_Toc199804750)

# **Appendix S1 Sputum Smear Experimental Method**

For sputum smear detection, the Ziehl-Neelsen staining method was employed, with detailed procedures as follows:

## S1.1 *Preparation of Sputum Smears*

- Use new, degreased, unscratched glass slides with a ground glass end. Label the ground glass end of each slide with a 2B pencil to indicate the laboratory number and specimen number. Only one sputum specimen should be smeared per slide.
- Carefully open the sputum container inside a biosafety cabinet to prevent aerosol generation or specimen spillage.
- Examine the specimen carefully. Using the broken end of a bamboo stick, select approximately 0.05–0.1 ml of caseous, purulent, or suspicious portions from the sputum. Evenly smear this material into a 10 mm × 20 mm oval sputum film on the right two-thirds of the front side of the slide. Allow the slide to air-dry horizontally for 30 minutes with the smear facing upward.

## S1.2 *Staining*

- **Fixation:** After the smear air-dries naturally, place the slide on a staining rack with at least 10 mm spacing between slides. Heat-fix by passing the slide through a flame four times within 5 seconds.
- **Primary Staining:** Flood the smear with carbol fuchsin stain. Gently heat the slide until steam appears, then stop heating and allow staining for 5 minutes. Ensure the smear remains completely covered with stain throughout; add additional stain if necessary. Do not allow the stain to boil.
- **Washing:** Gently rinse the slide with running water from one end to remove the stain, then drain excess water from the specimen.
- **Decolorization:** Drop decolorizing agent from the upper outer edge of the smear to completely cover it, and let decolorize for 1 minute.
- **Washing:** Gently rinse the slide with running water from one end to remove the decolorizing agent, then drain excess water from the slide.
- **Counterstaining:** Flood the smear with methylene blue counterstain and stain for 30 seconds.
- **Washing:** Gently rinse the slide with running water from one end to remove the counterstain, then drain excess water from the specimen. Allow the slide to air-dry completely before microscopic examination.

## S1.3 *Microscopic Examination*

- Examine slides using a binocular microscope with a 10× eyepiece.
- Place the stained and air-dried slide on the microscope stage with the sputum smear facing upward, securing it with mechanical stage clips.
- First, use the 40× objective. Adjust the mechanical stage to move the slide until the left end of the sputum smear is in view. Adjust the light intensity to an appropriate level and focus until cell morphology is clearly visible.
- Rotate away the 40× objective, place 1–2 drops of immersion oil directly on the slide over the smear, and use the 100× oil immersion objective for detailed examination.

## S1.4 *Criteria for Result Interpretation*

| **Report** | **Standard** |
| --- | --- |
| Acid-fast bacilli negative | Continuous observation of 300 distinct fields, with no acid-fast bacilli detected. |
| Acid-fast bacilli positive (reporting acid-fast bacilli) | 1–8 acid-fast bacilli per 300 fields. |
| Acid-fast bacilli positive (1+) | 3–9 acid-fast bacilli per 100 fields, with continuous observation of 300 fields. |
| Acid-fast bacilli positive (2+) | 1–9 acid-fast bacilli per 10 fields, with continuous observation of 100 fields. |
| Acid-fast bacilli positive (3+) | 1–9 acid-fast bacilli per field. |
| Acid-fast bacilli positive (4+) | ≥10 acid-fast bacilli per field. |

# **Appendix S2 Experimental methods for the isolation and cultivation of bacterial strains**

The strains were isolated and cultured using a simple mycobacteria method on solid culture. Sputum samples were pretreated with 4% NaOH and then inoculated into the acidic L-J medium. The cultures were incubated at a constant temperature of 37℃. The detailed procedures as follows:

## S2.1 *Mycobacterium isolation and culture steps*

- Transfer about 1 ~ 2 ml of the collected sputum specimen to the pretreatment tube in the biosafety cabinet;
- Depending on the characteristics of the specimen, add 4% NaOH solution about 1-4 times the volume of sputum into the pretreatment tube containing the sputum specimen;
- After screwing the screw cap of the pretreatment tube, place the pretreatment tube on a vortex oscillator for vortex oscillation for about 30s until the sputum specimen is fully liquefied. Let it stand at room temperature for 15 min;
- Unscrew the screw cap of L-J medium culture tube, check the solidified water at the bottom of the slant of the culture medium, if there is too much solidified water, discard the solidified water along the inner wall of the culture tube on the opposite side of the slant;
- Suck the sputum specimen after pretreatment with sterile pipette;
- Keep the slant level or the bottom of the culture medium slightly lower, and uniformly inoculate it on the slant of the acidic L-J medium, with 0.1-0.15ml (about 2-3 drops) for each culture medium. During inoculation, inoculate the first drop of liquid to the middle of the slant, and inoculate the second drop to the upper part of the culture medium;
- Screw the screw cap of the culture tube but avoid complete tightening;
- Gently rotate and lower the bottom of the culture tube, making the inoculated liquid evenly spread on the slant;
- Place the culture medium on a slant placing rack, and keeping the slant of the culture medium horizontally upward;
- Incubate the culture medium in a constant temperature incubator together with the slant placing rack, and set the temperature at 36±1℃; After 24 hours, tighten the screw cap of the culture tube, keep upright, and continue incubation at 36±1℃.

## S2.2 *Results reading*

- Observe the culture on Day 3 and Day 7 after inoculation, and then once a week until the 8th weekend. After each observation, record the observation results in the record book of culture results;
- Visual judgment: the typical colony morphology of MTB is: opaque pale yellow, rough, dry, protruding from the culture medium, with some in cauliflower patterns. If it is found the medium is liquefied, or mould is present, contamination is reported;
- Based on the preliminary visual judgment, record the results according to the Criteria of S2.2.1:

**S2.2.1 Criteria for result interpretation**

| **Growing status** | **Results** |
| --- | --- |
| No colony growth | Culture negative is reported |
| When the colony growth is less than 1/4 of the slant area | Actual colony numbers are reported |
| Colonies occupy 1/4 of the slant area | Report (1 +) |
| Colonies occupy 1/2 of the slant area | Report (2+) |
| Colonies occupy 3/4 of the slant area | Report (3+) |
| Colonies cover the slant of the medium | Report (4+) |

- If medium contamination is found, it is reported by area of contamination (see S2.2.2)

**S2.2. 2 Criteria for reported by area of contamination**

| **Contamination status** | **Results** |
| --- | --- |
| The contaminated bacteria have obvious boundaries not exceeding 1/4 of the slant area | Report (C1+) |
| The contaminated bacteria have obvious boundaries not exceeding 1/2 of the slant area | Report (C2+) |
| The contaminated bacteria have obvious boundaries not exceeding 3/4 of the slant area | Report(C3+) |
| The contaminating bacteria do not have a clear boundary or cover the slant of the medium | Report(C4+) |

# **Appendix S3 Experimental methods for the identification of microbial strains**

The strains were identified using P-Nitrobenzoic acid (PNB) and Thiophen-2-carboxylic acid hydrazide (TCH) identification medium. The detailed procedures as follows:

## S3.1 *Test procedures*

##### *Ⅰ. Preparation of bacterial solution*

- Add 1-2 drops of 10% Tween -80 aqueous solution into a bacteria-grinding bottle;
- Scrape a half inoculation ring or an inoculation ring (about 5-10 mg) of fresh colonies in the late logarithmic phase with the tip of a sterile straw or a flame-sterilized inoculation ring, and place it into a bacteria-grinding bottle;
- Screw the bottle cap, and oscillate the bottle on a vortex oscillator for 10-20 s;
- Let it stand for 5min, carefully open the bottle cap, add about 2ml of sterilized normal saline, and let it stand for a while to precipitate large substances in the bacterial liquid;
- Suck up the middle and upper part of the bacterial solution with a sterile pipette, about 1ml, transfer it to another sterile test tube, and compare the turbidity with a standard McFarland turbidimetric tube (MacFarland No.1);
- Gradually add sterilized normal saline dropwise until the turbidity of the bacterial solution is consistent with that in the standard McFarland No.1 tube, and 1mg/ml bacterial solution is obtained.

##### *Ⅱ. Inoculation*

- Bacterial liquid dilution: Add 2ml sterilized normal saline into a sterile test tube with a screw cap in a sterile manner for standby, one for each bacteria strain to be tested. The bacterial liquid is allowing to stand until particles or bacterial block are precipitated. Use 22 SWG standard inoculation ring to take 2 full rings of 1mg/ml of bacterial solution, translate it into 2ml of sterilized normal saline to dilute to 10^-2^mg/ml of bacterial solution;
- Inoculation: Take full ring (about 0.01 mL) of 10^–2^ mg/ml bacterial solution with a 22 SWG standard inoculation ring, and uniformly inoculate it onto the surface of PNB and TCH identification medium by streaking method. Attention should be paid to ensure that the bacterial solution was evenly dispersed on the inclined surface of the medium as possible.

##### *Ⅲ. Culture*

- The inoculated medium is placed upright in constant temperature culture at 36±1℃ for 4 weeks and the results are reported.

## S3.2 *Identification criteria*

Recording of results according to the criteria of S3.2.1.

S3.2.1 Criteria for identification of strain species

|  | **PNB** | **TCH** |
| --- | --- | --- |
| Mycobacterium tuberculosis(MTB) | - | + |
| Mycobacterium bovis | - | - |
| Nontuberculosis mycobacteria(NTM) | + | + |
| +: Grow on PNB or TCH;  -: Not Grow on PNB or TCH. | | |

# **Appendix S4 Antimicrobial susceptibility testing methods**

A phenotypic DST was conducted using the proportion method in the mycobacterium solid drug sensitivity test. This study included the assessment of isoniazid (H), rifampicin (R), ethambutol (E) and streptomycin (S). The detailed procedures as follows:

## S4.1 *Test procedures*

##### *Ⅰ. Preparation of bacterial solution*

- Add 1-2 drops of 10% Tween -80 aqueous solution into a bacteria-grinding bottle;
- Scrape a half inoculation ring or an inoculation ring (about 5-10 mg) of fresh colonies in the late logarithmic phase with the tip of a sterile straw or a flame-sterilized inoculation ring, and place it into a bacteria-grinding bottle;
- Screw the bottle cap, and oscillate the bottle on a vortex oscillator for 10-20 s;
- Let it stand for 5min, carefully open the bottle cap, add about 2ml of sterilized normal saline, and let it stand for a while to precipitate large substances in the bacterial liquid;
- Suck up the middle and upper part of the bacterial solution with a sterile pipette, about 1ml, transfer it to another sterile test tube, and compare the turbidity with a standard McFarland turbidimetric tube (McFarland No.1);
- Gradually add sterilized normal saline dropwise until the turbidity of the bacterial solution is consistent with that in the standard McFarland No.1 tube, and 1mg/ml bacterial solution is obtained.

##### *Ⅱ. Inoculation*

- Bacterial liquid dilution: Add 2ml sterilized normal saline into a sterile test tube with a screw cap in a sterile manner for standby, two for each bacteria strain to be tested. The bacterial liquid is allow to stand until particles or bacterial block are precipitated. Use 22 SWG standard inoculation ring to take 2 full rings of 1mg/ml of bacterial solution, translate it into 2ml of sterilized normal saline to dilute to 10^-2^mg/ml of bacterial solution. Dilute by 100 times in the same way to obtain 10^- 4^mg/ml bacterial solution;
- Inoculation: Take full ring (about 0.01 mL) of 10^–2^ mg/ml and 10^-4^mg/ml bacterial solution with 22 SWG standard inoculation rings respectively, and evenly inoculate them onto the control and drug-containing medium surfaces by streaking method. Attention should be paid to ensure that the bacterial solution was evenly dispersed on the inclined surface of the medium as possible. The final amounts of inoculated bacteria should be 10^-4^mg and 10^-6^mg.

Further detailed information on the Drug concentration in medium and flow chart test is present in S4.1.1 and S4.1.2.

**S4.1.1 Drug concentration in medium**

| Drug | Drug concentration in medium（μg/ml） |
| --- | --- |
| Isoniazid | 0.2 |
| Rifampicin | 40 |
| Ethambutol | 2 |
| Streptomycin | 4 |

Diluted to 10^-4^mg/ml bacterial suspension

Grinding bacteria

Fresh colonies 1-2 weeks after the appearance of visible colonies

Prepared to 1mg/ml bacterial suspension

Dilute 100 times

Diluted to 10^-2^mg/ml bacterial suspension

Dilute 100 times

Drug-free control medium

Inoculate 0.01ml

Inoculate 0.01ml

Drug-containing medium

**S4.1.2 Operation flow chart of proportional indirect method**

##### *Ⅲ. Culture*

- The inoculated medium is placed upright in constant temperature culture at 36±1℃ for 4 weeks and the results are reported.

## S4.2 *Results reading and resistance determination criteria*

##### *Ⅰ. Interpretation of drug sensitivity growth*

Recording of results according to the criteria of S4.2.1.

**S4.2.1** Criteria for results of Phenotypic Drug Susceptibility Testing

| **Colony growth** | **Reporting method** |
| --- | --- |
| No colony growth | Negative |
| Less than 50 colonies | Actual number of colonies |
| 50~100 colonies | 1+ |
| 100~200 colonies | 2+ |
| Mostly fusion(200~500 colonies) | 3+ |
| Fusion (more than 500 colonies) | 4+ |

##### *Ⅱ. Calculation and interpretation of percentage resistance*

Percentage of drug resistance(%) =$\frac{Number of colonies grown on drug-containing medium}{Number of colonies grown on control medium} \times100\%$

- If the percentage of resistance is greater than 1%, the test bacterium is considered to be resistant to the anti-tuberculosis drug.

## S4.3 *Quality control*

Strain H37Rv was employed as an internal control for isolation and cultivation of bacterial strains, identification of microbial strains, drug susceptibility testing. Quality control for each experimental batch (each experimental batch contains approximately 10 to 15 samples) was conducted using H37Rv, which was provided by the Chinese Center for Disease Control and Prevention (China CDC).

Throughout the testing period, the laboratory successfully passed the external quality assessment conducted by the National Center for Tuberculosis Control and Prevention, China Centers for Disease Control and Prevention. All laboratory activities, including reagent and media preparation, were carried out in strict accordance with standard operating procedures. Our reference standard is the*Technical Guidelines for Tuberculosis Prevention and Control in China*(<https://www.chinacdc.cn/jkyj/crb2/yl/fjh/jswj_fjh/202410/P020241010432930570191.pdf> ).

# **Appendix S5 Supplementary results**

## S5.1 *Logistic Regression Model Variable Assignment Table*

| **Variable** | | **Code** | | |
| --- | --- | --- | --- | --- |
| Dependent variable | | Drug susceptibility=0 | Drug resistance=1 |  |
| Independent variable |  |  |  |  |
|  | Sex | Male=0 | Female=1 |  |
|  | Therapeutic Category | Newly treatment TB=0 | Retreatment TB=1 |  |
|  | Diabetes | No=0 | Yes=1 |  |
|  | Floating Population | No=0 | Yes=1 |  |
|  | Retiree | No=0 | Yes=1 |  |
|  | Farmer | No=0 | Yes=1 |  |
|  | Smear Result | Negative=0 | Positive=1 |  |
|  | HIV | Negative=0,0 | Positive=1,0 | Not Provide=0,1 |

## S5.2 The Hosmer- Lemeshow goodness-of-fit test result of the logistic regression

| **Model** | ***χ^2^*** | ***P*** |
| --- | --- | --- |
| MDR | 15.0609 | 0.0580 |
| Resistance to H | 2.7560 | 0.9487 |
| Resistance to R | 3.7824 | 0.8762 |
| Resistance to E | 5.7166 | 0.6789 |
| Resistance to S | 5.4974 | 0.7033 |

## S5.3 The results of the multivariate logistic regression analysis

|  | | | β | S.E | Wals | P | OR | OR 95% C.I. | |
| --- | --- | --- | --- | --- | --- | --- | --- | --- | --- |
|  |  |  |  |  |  |  |  | Lower | Upper |
| MDR |  |  |  |  |  |  |  |  |  |
|  | Constant |  | -2.5723 | 0.4975 | 26.7381 | ＜0.0001 | 0.0764 | - | - |
|  | Age |  | -0.0110 | 0.0095 | 1.3331 | 0.2483 | 0.9891 | 0.9709 | 1.0077 |
|  | Sex |  | -0.3702 | 0.3298 | 1.2597 | 0.2617 | 0.6906 | 0.3618 | 1.3182 |
|  | Farmer |  | 0.3709 | 0.5300 | 0.4897 | 0.4840 | 1.4491 | 0.5128 | 4.0951 |
|  | Retiree |  | 0.0163 | 0.5495 | 0.0009 | 0.9764 | 1.0164 | 0.3462 | 2.9842 |
|  | Floating Population |  | -0.0870 | 0.3117 | 0.0779 | 0.7802 | 0.9167 | 0.4976 | 1.6888 |
|  | Diabetes |  | -0.0122 | 0.4953 | 0.0006 | 0.9804 | 0.9879 | 0.3742 | 2.6080 |
|  | HIV | Negative | - | - | 3.0006 | 0.2231 | - | - | - |
|  |  | Positive | 0.7515 | 0.5700 | 1.7383 | 0.1874 | 2.1201 | 0.6937 | 6.4793 |
|  |  | Not Provide | 0.5895 | 0.4882 | 1.4581 | 0.2272 | 1.8031 | 0.6926 | 4.6943 |
|  | Therapeutic Category |  | 0.5739 | 0.3468 | 2.7388 | 0.0979 | 1.7752 | 0.8996 | 3.5032 |
|  | Smear Result |  | -0.2168 | 0.3423 | 0.4013 | 0.5264 | 0.8051 | 0.4116 | 1.5746 |
| Resistance to H |  |  |  |  |  |  |  |  |  |
|  | Constant |  | -2.3426 | 0.3360 | 48.6107 | ＜0.0001 | 0.0961 | - | - |
|  | Age |  | 0.0074 | 0.0059 | 1.5614 | 0.2115 | 1.0074 | 0.9958 | 1.0191 |
|  | Sex |  | -0.1065 | 0.2043 | 0.2717 | 0.6022 | 0.8990 | 0.6023 | 1.3418 |
|  | Farmer |  | 0.2566 | 0.3538 | 0.5260 | 0.4683 | 1.2925 | 0.6461 | 2.5858 |
|  | Retiree |  | -0.5290 | 0.3351 | 2.4919 | 0.1144 | 0.5892 | 0.3055 | 1.1363 |
| **Continued on next page** | | | | | | | | | |
| **Continued from previous page** | | | | | | | | | |
|  | Floating Population |  | 0.1388 | 0.2015 | 0.4749 | 0.4907 | 1.1489 | 0.7741 | 1.7053 |
|  | Diabetes |  | 0.3668 | 0.2834 | 1.6748 | 0.1956 | 1.4432 | 0.8280 | 2.5153 |
|  | HIV | Negative | - | - | 0.0272 | 0.9865 | - | - | - |
|  |  | Positive | 0.0503 | 0.4968 | 0.0102 | 0.9194 | 1.0516 | 0.3971 | 2.7845 |
|  |  | Not Provide | 0.0478 | 0.3570 | 0.0179 | 0.8935 | 1.0490 | 0.5210 | 2.1119 |
|  | Therapeutic Category |  | 0.5293 | 0.2320 | 5.2047 | 0.0225 | 1.6977 | 1.0774 | 2.6750 |
|  | Smear Result |  | -0.3655 | 0.2266 | 2.6012 | 0.1068 | 0.6939 | 0.4450 | 1.0818 |
| Resistance to R |  |  |  |  |  |  |  |  |  |
|  | Constant |  | -1.9411 | 0.4102 | 22.3918 | ＜0.0001 | 0.1435 | - | - |
|  | Age |  | -0.0142 | 0.0080 | 3.1601 | 0.0755 | 0.9859 | 0.9707 | 1.0015 |
|  | Sex |  | -0.3256 | 0.2703 | 1.4505 | 0.2285 | 0.7221 | 0.4251 | 1.2266 |
|  | Farmer |  | 0.3870 | 0.4609 | 0.7051 | 0.4011 | 1.4726 | 0.5967 | 3.6345 |
|  | Retiree |  | -0.0756 | 0.4701 | 0.0258 | 0.8723 | 0.9272 | 0.3690 | 2.3299 |
|  | Floating Population |  | -0.1338 | 0.2573 | 0.2704 | 0.6031 | 0.8748 | 0.5283 | 1.4485 |
|  | Diabetes |  | 0.1171 | 0.4007 | 0.0854 | 0.7701 | 1.1242 | 0.5126 | 2.4654 |
|  | HIV | Negative | - | - | 1.1184 | 0.5717 | - | - | - |
|  |  | Positive | 0.4912 | 0.5102 | 0.9268 | 0.3357 | 1.6342 | 0.6012 | 4.4420 |
|  |  | Not Provide | 0.2142 | 0.4442 | 0.2326 | 0.6296 | 1.2389 | 0.5187 | 2.9590 |
|  | Therapeutic Category |  | 0.6358 | 0.2857 | 4.9537 | 0.0260 | 1.8886 | 1.0789 | 3.3060 |
|  | Smear Result |  | -0.2341 | 0.2856 | 0.6717 | 0.4124 | 0.7913 | 0.4521 | 1.3850 |
| Resistance to E |  |  |  |  |  |  |  |  |  |
|  | Constant |  | -2.9976 | 0.5604 | 28.6123 | ＜0.0001 | 0.0499 | - | - |
|  | Age |  | -0.0032 | 0.0106 | 0.0930 | 0.7604 | 0.9968 | 0.9764 | 1.0176 |
| **Continued on next page** | | | | | | | | | |
| **Continued from previous page** | | | | | | | | | |
|  | Sex |  | -0.1283 | 0.3438 | 0.1392 | 0.7091 | 0.8796 | 0.4484 | 1.7255 |
|  | Farmer |  | 0.2920 | 0.6189 | 0.2226 | 0.6371 | 1.3391 | 0.3981 | 4.5038 |
|  | Retiree |  | 0.7971 | 0.5232 | 2.3209 | 0.1276 | 2.2190 | 0.7958 | 6.1875 |
|  | Floating Population |  | 0.0784 | 0.3479 | 0.0508 | 0.8216 | 1.0816 | 0.5469 | 2.1390 |
|  | Diabetes |  | -1.6869 | 1.0235 | 2.7164 | 0.0993 | 0.1851 | 0.0249 | 1.3759 |
|  | HIV | Negative | - | - | 0.4635 | 0.7931 | - | - | - |
|  |  | Positive | -0.3687 | 1.0403 | 0.1256 | 0.7230 | 0.6916 | 0.0900 | 5.3138 |
|  |  | Not Provide | 0.3114 | 0.5489 | 0.3218 | 0.5705 | 1.3653 | 0.4656 | 4.0036 |
|  | Therapeutic Category |  | 0.1929 | 0.4377 | 0.1943 | 0.6594 | 1.2128 | 0.5143 | 2.8598 |
|  | Smear Result |  | -0.4535 | 0.3655 | 1.5392 | 0.2147 | 0.6354 | 0.3104 | 1.3007 |
| Resistance to S |  |  |  |  |  |  |  |  |  |
|  | Constant |  | -2.2702 | 0.3258 | 48.5681 | ＜0.0001 | 0.1033 | - | - |
|  | Age |  | 0.0077 | 0.0057 | 1.8002 | 0.1797 | 1.0077 | 0.9965 | 1.0191 |
|  | Sex |  | -0.2688 | 0.2008 | 1.7913 | 0.1808 | 0.7643 | 0.5156 | 1.1330 |
|  | Farmer |  | -0.0656 | 0.3515 | 0.0348 | 0.8520 | 0.9365 | 0.4703 | 1.8650 |
|  | Retiree |  | -0.1261 | 0.2962 | 0.1813 | 0.6703 | 0.8815 | 0.4932 | 1.5754 |
|  | Floating Population |  | 0.1338 | 0.1957 | 0.4677 | 0.4941 | 1.1432 | 0.7790 | 1.6775 |
|  | Diabetes |  | 0.4604 | 0.2626 | 3.0730 | 0.0796 | 1.5846 | 0.9471 | 2.6513 |
|  | HIV | Negative | - | - | 4.2800 | 0.1177 | - | - | - |
|  |  | Positive | -0.9972 | 0.7376 | 1.8277 | 0.1764 | 0.3689 | 0.0869 | 1.5659 |
|  |  | Not Provide | 0.4885 | 0.3208 | 2.3191 | 0.1278 | 1.6299 | 0.8692 | 3.0564 |
|  | Therapeutic Category |  | 0.4561 | 0.2297 | 3.9430 | 0.0471 | 1.5779 | 1.0059 | 2.4752 |
|  | Smear Result |  | -0.3518 | 0.2207 | 2.5398 | 0.1110 | 0.7034 | 0.4564 | 1.0842 |
| S: Streptomycin, H: Isoniazid, R: Rifampicin, E: Ethambutol. | | | | | | | | | |
